# Extracellular Vesicles in Musculoskeletal Regeneration: Modulating the Therapy of the Future

**DOI:** 10.3390/cells11010043

**Published:** 2021-12-24

**Authors:** Hugo Abreu, Elena Canciani, Davide Raineri, Giuseppe Cappellano, Lia Rimondini, Annalisa Chiocchetti

**Affiliations:** 1 Department of Health Sciences, Interdisciplinary Research Center of Autoimmune Diseases—IRCAD, Università del Piemonte Orientale, 28100 Novara, Italy; hugo.abreu@uniupo.it (H.A.); davide.raineri@uniupo.it (D.R.); giuseppe.cappellano@med.uniupo.it (G.C.); lia.rimondini@med.uniupo.it (L.R.); annalisa.chiocchetti@med.uniupo.it (A.C.); 2 Center for Translational Research on Autoimmune and Allergic Diseases—CAAD, Università del Piemonte Orientale, 28100 Novara, Italy

**Keywords:** regenerative medicine, mesenchymal stem/stromal cells, extracellular vesicles, biomaterials

## Abstract

Tissue regeneration is a hot topic in health sciences, particularly because effective therapies promoting the healing of several cell types are lacking, specifically those of the musculoskeletal system. Mesenchymal Stem/Stromal Cells (MSCs) have been identified as crucial players in bone homeostasis, and are considered a promising therapy for diseases such as osteoarthritis (OA) and Rheumatoid Arthritis (RA). However, some known drawbacks limit their use, particularly ethical issues and immunological rejections. Thus, MSCs byproducts, namely Extracellular Vesicles (EVs), are emerging as potential solutions to overcome some of the issues of the original cells. EVs can be modulated by either cellular preconditioning or vesicle engineering, and thus represent a plastic tool to be implemented in regenerative medicine. Further, the use of biomaterials is important to improve EV delivery and indirectly to modulate their content and secretion. This review aims to connect the dots among MSCs, EVs, and biomaterials, in the context of musculoskeletal diseases.

## 1. Clinical Background

Musculoskeletal disorders (MSDs) are conditions that can limit bodily movement through injury or pain in tissues of the musculoskeletal system, such as muscles, bones, and joints. The most common diseases are osteoarthritis (OA), rheumatoid arthritis (RA), neck and low back pain, tendinitis, carpal tunnel syndrome, fibromyalgia, and bone fractures [1,2,3]. Risk factors include occupation, lifestyle, and family history. However, since the risk of developing MSDs greatly increases with age, their prevalence increases with the rising average life expectancy [1]. The severity of MSDs ranges from mild pain and discomfort, which interferes with everyday activities, to total impairment of movement. For this reason, early diagnosis and treatment may help ease symptoms and improve long-term quality of life [4,5]. In particular, OA is defined as a chronic joint inflammatory disease that affects all joint tissues of the musculoskeletal system, impacting mainly the hip, hand and knee articulations [6], involving tissues such as the infrapatellar fat pad and meniscus [7,8]. Patients bearing OA usually show synovial inflammation, calcified ligaments, subchondral bone sclerosis, osteophyte formation, and cartilage deterioration [9]. It is estimated that around 250 million people worldwide are suffering from OA [6]. Conversely, RA is an autoimmune inflammatory disorder characterized by synovial joint inflammation and swelling, estimated to affect up to 1% of the population worldwide [10]. Both these MSDs are common causes of permanent disability among the elderly population [11].

There is no cure for MSDs OA and RA; the only available clinical options aim to reduce the impact of the most common symptoms caused by inflammation, especially pain [5,12,13]. These conditions typically progress towards permanent joint damage, and treatments are intended to slow disease evolution. Currently, non-steroidal anti-inflammatory drugs (NSAIDs) are frequently administered to manage the overall disabilities caused OA [5]. This strategy can in some severe cases be coupled with surgery to replace severely damaged joints. However, considering the multiple complications for the patient associated with this approach, as well as its ineffectiveness in restoring tissue function and movement, the need for novel and effective therapies is clear [5].

New approaches to regenerating musculoskeletal tissues are now emerging. One rapidly-growing strategy to treat damaged tissue, and diseases such as OA and RA, is regenerative therapy; this has the advantage of being a multifactorial approach that combines biomaterial design mimicking the natural extracellular matrix (ECM) of the tissues, these materials being loaded with autologous cells, bioactive molecules and growth factors to stimulate and improve the function of the target tissue [14].

Immunoengineering is a branch of regenerative medicine that focuses on the immune system and that aims to modify the cellular response in order to facilitate tissue reconstruction [15]. In particular, joint diseases such as OA and RA have been targeted with biomaterials and anti-cytokine treatments as a potential innovative therapy [16]. Considering the lack of effectiveness of the currently administered anti-inflammatory drugs in both OA and RA, the current task for researchers and clinicians in the musculoskeletal regeneration field, is to develop and implement more effective therapies. This may occur through the use of biomaterials and the employment of genetic engineering in order to modulate cell behavior.

Within this scenario, the review explores the current research trends regarding tissue regeneration in MSDs, focusing on Extracellular Vesicles (EVs) as future strategies for musculoskeletal system repair through cell therapy, biomaterial science, and immunoengineering. It focuses on the byproducts of Mesenchymal Stem/Stromal Cells (MSCs), more specifically EVs.

## 2. MSCs: Function and Mechanism of Action upon Tissue Damage

**Figure 1:**
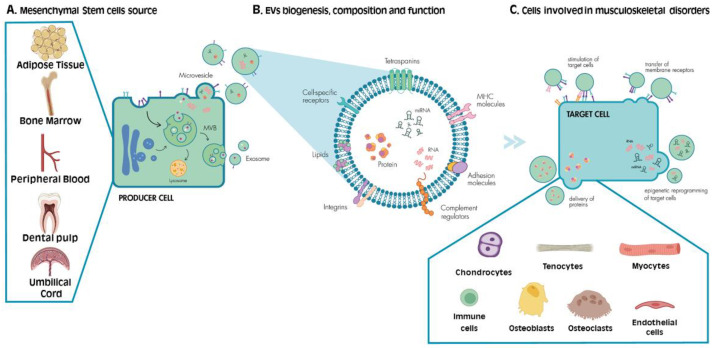
Cell-to-cell Communication via EVs in the Musculoskeletal System. (**A**) MSCs can be found in the bone marrow, adipose tissue, dental pulp, peripheral blood, umbilical cord, and among other tissues. (**B**) MSCs release EVs containing proteins, lipids, and nucleic acids (DNA and miRNA) to the surrounding environment. EVs can be formed either through plasma membrane budding (microvesicles) or through an endosomal route (exosomes). (**C**) They express surface markers that interact with the membrane receptors of the target cells (chondrocytes, myocytes, osteoclasts, osteoblasts, immune cells, tenocytes, among others) impacting the vesicle uptake and cargo delivery or directly stimulating and/or reprogramming the target cell. Created with BioRender.com.

MSCs or multipotent mesenchymal stromal cells, as recommended by the International Society for Cellular Therapy (ISCT) [17], are fibroblast-like adult multipotent progenitor cells that possess the ability to self-renew and differentiate in vitro into several cell types [18], mainly into osteoblasts, adipocytes and chondrocytes. MSCs can be harvested from numerous tissues, including bone marrow (BM-MSCs) and other parts of the bone (periosteum and endosteum), adipose tissue (AT-MSCs) such as the infrapatellar fat pad (IFP-MSCs), umbilical cord (UC-MSCs), peripheral blood, and oral tissues [19,20,21,22,23] (Figure 1A).

Oryan et al. have reviewed and grouped the advantages and disadvantages of each type of MSCs, and report that although AT-MSCs achieve higher cell viability, BM-MSCs are more stable in culture and more prone to differentiate into osteoblasts, thus being preferable for transplantation in cases of bone fractures [20]. The therapeutic delivery of BM-MSCs at bone fracture sites is a strategy worthy of consideration, owing to their osteogenic and chondrogenic capabilities [19].

In physiological conditions, MSCs naturally migrate to the site of bone injury/damage, where osteogenesis—i.e., differentiation into osteoblasts (cells capable of forming bone)—occurs [24]. This transition from MSCs into osteoblasts begins with the commitment towards the osteogenic/chondrogenic lineages through the Wingless-int (Wnt) pathway [25]. During this process, there is a natural upregulation of several transcription factors including Distal-less homeobox 5 (DLX5), Osterix (OSX), and Runt-related transcription factor 2 (RUNX2) [25,26,27] aimed at triggering the differentiation cascade. In turn, this leads to the overexpression of typical osteoblast-related proteins, namely alkaline phosphatase (ALP) that promotes collagen type I (COL1A1) deposition, essential for proper bone formation [28].

After bone damage/fracture, the inflammation process starts [29], with an increased secretion of chemotactic factors and cytokines that mediate bone regeneration. Both innate (macrophages) and adaptive (lymphocytes) immunity act upon injury by phagocyting necrotic tissues and secreting growth factors and cytokines, which collectively contribute to bone repair [20].

In this phase, the MSCs’ secretome can promote polarization of macrophages into the M2 anti-inflammatory and the M1 pro-regenerative phenotypes, through the nuclear factor kappa-light-chain-enhancer of activated B cells (NF-κB) and the signal transducer and activator of transcription 3 (STAT3) pathways [30,31,32]. In addition, the expression of tumor necrosis factor-alpha (TNF-α) can be reduced through MSCs transplantation; since high levels of TNF-α have a proapoptotic effect in osteoblasts, this should improve bone formation [33]. TNF-α also inhibits the expression of RUNX2 and Osterix, suggesting MSCs could facilitate the host osteoblast formation by restoring normal levels of the two essential transcription factors in osteogenesis [34]. Further, MSCs have been reported to contribute to blood vessel formation, known to be crucial in bone formation and repair [20]. In summary, MSCs are promising for therapeutic applications due to their immunomodulatory, angiogenic, cell recruitment, antiapoptotic, and differentiation effects [20,24].

In MSDs, such as OA disease, MSCs play a pivotal role in immunomodulation, modifying their phenotype in response to molecules produced by damaged tissues, both in acute and chronic phases of the disease [35]. Zhao et al. reported that MSCs are able to regulate macrophages polarization, promoting the healing process. This process brings an overall improvement of inflammation with an increase in interleukin -10 (IL-10) levels and a decrease of IL-12 and IL-1β and a contemporaneous augmentation of the phagocytes’ activity [35]. Clinically, MSCs demonstrated to be effective in reducing pain for 5 years after joint injection of MSCs in OA patients [5].

Various bone injuries and anomalies reduce the natural healing process, pointing up the importance of developing new strategies for bone regeneration [36]. MSCs have thus emerged as a promising candidate therapy for MSDs [36]. MSCs can be delivered by systemic injection, as used to treat osteoporosis [37], by local/direct injection, as in the case of nonunion fractures [38,39,40] or administered with hydrogels or scaffolds, such as decellularized ECM, as in the case of large bone defects [20].

Much research has studied how MSCs resolve impaired bone regeneration conditions, mainly resorting to in vivo animal models [41]. A first study attempted to inject allogeneic BM-MSCs into rats, locally or systemically, to promote fracture healing [37]. Although both treatments showed significant improvement in tissue healing, without triggering an adverse immune response, the authors point out that, in cases of deeper multiple fractures, as expected in osteoporotic patients, the systemic injection might be more beneficial [37]. Other researchers have focused on this approach, paying special attention to MSCs’ migration and homing for effective correction of bone lesions, with promising results [42,43,44]. Regarding nonunion fractures, various studies have resorted to MSCs to stimulate bone regeneration, all with fairly positive results, healing times ranging from 4 weeks to 10 months (reviewed by Fayaz et al. [45]).

Lastly, in an attempt to increase treatment efficacy, MSCs have been combined with biomaterials. These scaffolds ideally promote MSCs’ local delivery and viability, while stimulating osteogenesis [24]. Freitas et al. reviewed the properties, advantages, and disadvantages of current materials in this field: ceramic biomaterials—commonly consisting of calcium phosphate (CaP) in the form of hydroxyapatite (HA), β-tricalcium phosphate (β-TCP), or a combination of both, known as biphasic calcium phosphate (BCP); polymers, both natural and synthetic; composites, i.e., a mix of polymeric biomaterials and ceramics; and nanoparticles (NP) [24]. Additionally, graphene and metals, such as titanium and tantalum, are also valid options thanks to their biocompatibility, and their stimulation of proliferation and differentiation [46]. MSCs are already being implemented in clinical trials, in oral and maxillofacial surgery [47,48,49] and in large bone defects [20,50].

Currently, nearly 400 clinical trials focused on using MSCs to treat pathologies related to multiple organs and cell lineages have been completed, and many more are listed as ongoing (http://www.clinicaltrials.gov/ (accessed on 21 December 2021)). These studies focus on a wide range of diseases, including autoimmune diseases such as RA [51], type 1 and 2 diabetes mellitus [52], multiple sclerosis [53] and systemic lupus erythematosus [54], as well as OA [55], graft versus host disease [56], chronic kidney disease [57], idiopathic pulmonary fibrosis [58], cirrhosis [59], acute myocardial infarction [60] and COVID-19 [61].

Some clinical trials report improvements in OA treatment through the use of MSCs [55]. Centeno et al. combined BM-MSCs with bone marrow aspirates and platelet lysates, improving chondral and meniscus volume while also greatly reducing pain in 60% of patients, and reducing to one-tenth the need for replacement surgery [62]. There are also several active and competed clinical studies regarding RA. For example, Wang et al. administered UC-MSCs intravenously to patients affected by RA (4 × 10^4^ cells/injection), together with the common disease-modifying anti-rheumatic drugs (DMARDs) treatment, at intervals of 3, 6 or 8 months. They report an improvement in quality of life and reduced joint swelling and pain, compared with control groups (treated with DMARDs alone), assessed via the disease activity score 28 (DAS28) parameter and the Health Assessment Questionnaire (HAQ) [63]. Using a different approach, Park et al. opted for altering the dosage of UC-MSCs in a single intravenous injection. In this study, patients who received the higher concentration of cells (1 × 10^8^ cells) showed lower DAS28 and visual analog scale (VAS) scores, indicative of therapy efficacy [64]. Of note, clinical trials using MSCs as a potential treatment for RA have been considered safe, no toxicity or severe adverse effects being reported thus far [51].

However, drawbacks including ethical issues, immunological rejection, costs, and low quantity of cells harvested, still need to be overcome in order to apply MSCs’ transplantation as a therapy [20,65]. It is also reported that MSCs can cause serious secondary effects. In one such case unwanted differentiation after transplantation into the brain of a child with Ataxia Telangiectasia caused tumors [66]. In order to counteract some of the above problems, the use of MSCs secretome, and more specifically of MSC-derived EVs, has increasingly been employed over recent years [67].

Interestingly, MSCs’ secretome can mimic most of the effects of the cells themselves [67]: it contains paracrine products of MSCs’ metabolism that promote wound healing by increasing proliferation and differentiation [68] and exerts immunomodulation [69], cell recruitment [70], pro-angiogenic [69] and pro-survival [71] functions, indicating that this cell-free approach may represent an alternative therapy [67]. This thanks to its composition, essentially comprising growth factors (GF) (Epidermal-GF (EGF), Fibroblast- GF(FGF), Hepatocyte-GF (HGF), Insulin-like-GF (IGF), Platelet-Derived-GF (PDGF), interleukins (IL-6, -8, -10), matrix metallopeptidases (MMP )-1, -2, -3, -7) and MMP inhibitors (TIMP-1, -2), angiogenic factors (Vascular-Endothelial-GF(VEGF), Angiogenic Factors Angiopoietin (ANG), chemoattracting proteins (Chemokine (C-C motif) ligand 5 CCL5/RANTES, monocyte chemoattractant protein 1 (MCP-1), adhesion molecules (intercellular adhesion molecule(ICAM), vascular cell adhesion molecule (VCAM), and immunoregulators (Transforming Growth Factor-β (TGF-β), Indoleamine 2,3-dioxygenase (IDO) [14,54]. These molecules are either free, outside cell boundaries, or encapsulated in EVs [67].

## 3. EVs: Biogenesis and Function

Studies focusing on cell-to-cell communication have employed body fluids, for example saliva, urine, blood, and breast milk, to show that all cell types can release lipidic bilayer vesicles, now generically labeled as EVs [72,73]. It is known that EVs naturally carry proteins, lipids, and nucleic acids, also possessing the potential to be bioengineered as drug delivery systems [74,75] (Figure 1B). Depending on both EVs cargo and the target cell type, EVs can influence various cellular processes, more specifically proliferation, differentiation, senescence, and apoptosis, while also impacting immunomodulation, blood coagulation, and angiogenesis [76,77].

Currently, the International Society of Extracellular Vesicles (ISEV) recommends making a distinction among three main types of EVs, based on size and release mechanism: apoptotic bodies, microvesicles, and exosomes [73]. Apoptotic bodies are the largest of these, ranging from 50 nm to 5 μm. They form by blebbing of the plasma membrane in cases of programmed cell death [78]. Microvesicles (MVs) are shed from budding of the plasma membrane, and their size is between 50 nm and 1 μm. Lastly, exosomes are typically less than 150 nm in diameter, and are the result of the fusion of multivesicular bodies (MVBs) with the plasma membrane, releasing intraluminal vesicles (ILVs) into the surrounding microenvironment, hence the term exosomes [73].

Exosome biogenesis starts with the formation of early endosomes, through endocytosis of extracellular components [75]. Budding of the membrane to capture proteins, lipids, and other molecules causes the formation of ILVs, small bodies the size of exosomes, that collect inside endosomes, leading to their maturation into MVBs [75]. ILVs are mainly created by action of the endosomal sorting complex required for transport (ESCRT), a family of four protein complexes, ESCRT-0, -I, -II and -III. MVBs can be degraded by fusing with lysosomes, or may in turn join the plasma membrane, so that their content is released outside the cellular compartment [75]. Some other proteins, specifically those frequently used as exosome markers, also contribute to exosome biogenesis, including CD63, MHC class II, tumor Susceptibility 101 (TSG101), ALG-2-interacting protein X (ALIX), syndecan, syntenin and Heat Shock Protein 70 (HSP70) [75].

Cells under various stimuli can increase or decrease EVs secretion in vitro [72,75]. In particular cases, irradiation [79], hypoxia [80] and other chemical stresses [81,82] may cause cells to boost EVs production and release. Whether this is a way to expel unwanted molecules produced under these stress conditions, or whether it is a signal to neighboring or distant cells, is still a matter of debate [73].

After release, EVs can be incorporated by target cells by direct fusion with the plasma membrane or by endocytosis, either through pinocytosis or by phagocytosis, dependently or independently of clathrin or caveolin, or lipid-raft mediated [75,83]. In general, it is thought that this mechanism is dependent on the recognition of specific surface markers on the vesicle membrane by the cellular membrane [75,84]: tetraspanins present on the EVs surface, such as CD9 and CD81, have a known positive effect in cell adhesion and in viral/parasitic internalization, which may explain their role as vesicle membrane proteins [83,85]. Other protein families have been associated with the EV uptake mechanisms, including integrins (αv, β3 and Lymphocyte function-associated antigen 1(LFA-1 [85]), proteoglycans (HSPGs [86]) and lectins (Dendritic Cell-Specific Intercellular adhesion molecule-3-Grabbing Non-integrin(DC-SIGN) [87] and DEC-205 [88]), since using antibodies against these molecules heavily impacts vesicle internalization in vitro [83]. It is also considered that the preferential EV uptake method may be associated with the cell type and its physiological state [89]. While some cases of specific/preferential uptake of EVs by a certain cell type have been described [87,90], there is still no consensus as to whether the internalization process is indeed cell-type specific [83].

On their surface, EVs can also display several molecules that directly affect a target cell without the need for uptake. Immunoglobulins, complement proteins, coagulation factors, cytokines, enzymes, and DNA have been detected in association with the vesicle membrane [91]. In the context of RA, Cloutier et al. demonstrated that synovial fluid contains platelet-derived EVs displaying immunoglobulins, antigens, and complement proteins as immune complexes capable of exerting a pro-inflammatory signal, in the presence of neutrophils [92]. Lastly, EVs can also function as antigen-presenting vehicles, as shown by Raposo et al., who reported that murine and human B lymphocytes release vesicles containing MHC class II molecules that can activate a specific T cell response [93]. Antigen-presenting EVs can also stimulate both T cells and macrophages phenotype shift towards Tregs and M2, respectively, which diminishes the length of the inflammatory phase in musculoskeletal regeneration [94].

Immune cells were shown to be able to transfer membrane receptors to other cells. For example, leukocyte-derived EVs can transfer monocyte/macrophage tissue factor to platelets [95]. Conversely, platelet-specific adhesion molecules can be relocated to hematopoietic cells through the action of EVs [96]. Other functions of EVs include epigenetic reprogramming of the target cells by altering the DNA methylation patterns, histone modifications and non-coding RNA post-transcriptional editing [97] (Figure 1C).

## 4. MSC-Derived EVs as Novel Therapies: Advantages and Disadvantages

Recent studies have also compared the ability of MSC-derived EVs to mimic the effect of their parent cells [98], and have concluded that MSC-EVs can exert similar effects to MSCs, in terms of injury repair and tissue regeneration [99,100,101,102], anti-inflammatory profile [103], cell proliferation and migration [102,104], and promoting collagen synthesis [105] and angiogenesis [102,104,105]. This indicates that MSC-EVs can replace the original cells as a therapeutic tool, eliminating some of the adverse effects of MSCs [98,106]. Indeed, the low immunogenicity and the low toxicity of EVs compared with MSCs are an important advantage of using these vehicles, together with their offering more stable storage, high stability in circulation, easier large-scale production, and superior biocompatibility [107,108]. Additionally, in contrast with MSCs, EVs can penetrate the blood-brain barrier, thus overcoming the first pass effect typical of some drug treatments [108].

MSCs produce EVs that express chemokines receptors able to facilitate targeting with several cell types also in damaged tissues [98]. Furthermore, new technologies, such as decorating EVs surface with binding proteins, are being developed, in order to increase the delivery efficiency of EVs to the target cells, improving cell-to-cell communication [98,109]. These strategies could be very useful to modulate the cellular communication in the musculoskeletal field.

For example, regarding musculoskeletal regeneration, Qin et al. evaluated the impact of MSC-EVs in osteoblasts’ biological processes: in this study, culturing human osteoblasts (hFOB 1.19 cell line) with MSC-EVs led to a similar degree of differentiation as exposing the cells to fresh osteoblast culture medium [110]. In addition, the expression of osteogenesis-related genes such as ALP, osteocalcin (OCN), osteopontin (OPN) and RUNX2 in the EVs-treated condition was similar to the hFOBs culture in a commercially available complete osteogenesis media [110]. Conversely, MSC-EVs also impair osteoclast formation in vivo, as reported by Hu et al., in an osteoporotic mouse model [111]. The authors injected human UC-MSC-derived EVs through the tail vein of the osteoporotic mice and noted a decrease in osteoclast number on the trabecular bone surface, coupled with increased number of osteoblasts, compared to the control group [111].

Furthermore, it has been reported in a rat osteochondral defect model that MSC-EVs promote cellular proliferation and migration in both cartilage and synovium, while also stimulating ECM deposition [112]. Additionally, rats treated with MSC-EVs exhibited an increase in M2 macrophage infiltration in cartilage and synovium, accompanied by a reduction of pro-inflammatory cytokines’ expression and M1 polarization, which leads to a regenerative immune profile [112]. On another study, muscle tissue regeneration was achieved by Nakamura et al.: the authors reported that in vitro culture of C2C12 cells (mouse myoblast cell line) with MSC-EVs promotes cell proliferation and differentiation, through the increase of nuclei number, fusion index and myogenic markers expression [113]. Moreover, the same authors show that in a mouse model of cardiotoxin-induced muscle injury, MSC-EVs locally administered at the injury site reduce fibrosis and increase angiogenesis, hence improving muscle regeneration [113]. Lastly, Chen et al. tested the ability of EVs to repair tendon damage. In a study performed in rabbits that underwent Achilles tendon repair surgery, the group administered with AT-MSC-EVs evidenced greater tenocyte proliferation and migration, together with mechanical stress resistance improvement of the tendon, compared to the control group [114] (Figure 1C).

However, therapies with EVs must be approached with caution, since their effects have yet to be fully characterized. Information currently available has led researchers to disagree about the tumor induction capacity of MSC-EVs. Some data indicates that MSC-EVs can inhibit tumor cell proliferation and induce dormancy and apoptosis [115,116,117]; conversely, MSC-EVs are also reported to confer drug resistance and support cancer cell growth and metastatization [118,119] (reviewed by Zhang et al. [120]).

Further, there is no protocol standardization regarding MSC culture and EV isolation in the clinical field. The commonly employed use of fetal bovine serum (FBS) in MSC expansion may cause the unwanted co-isolation of bovine EVs together with MSC-EVs [121], whereas completely removing the serum can impact EV quantity and cargo [122]. Using different EV isolation techniques—ultracentrifugation, differential centrifugation, commercial isolation kits, size-exclusion chromatography, iodixanol density gradient [123], among others—with differing yields and purification efficiencies may also contribute to the disparity of results reported in the literature [98]. Indeed, these different approaches may lead to false conclusions, since bovine-EVs from FBS (and other contaminants) may mask the effects of the target EVs. As an example, FBS-EVs have been associated with increased migration of A549 epithelial adenocarcinoma cell line, compared with EVs that are FBS “depleted” by extended centrifugation, with an estimated removal of 95% of FBS-EVs [121].

## 5. Impact of Biomaterials in MSC-Derived Extracellular Vesicles

As mentioned above concerning MSCs, the delivery of EVs is also an important factor to be taken into consideration. The so-called ‘Naked EVs’ administration, i.e., without any coating, can lead to unspecific binding to non-target organs, which in turn causes side effects and reduces the concentration at the injury site, hampering treatment efficacy [124]. Conjugation with biomaterials is thus considered a viable strategy to ensure correct targeting, preservation, and controlled release of the treatment [125]. Currently, the most widely studied coadjutants in EV delivery are hydrogels and scaffolds through vesicle encapsulation [125].

Hydrogels are cross-linked polymer chain networks with hydrophilic properties, containing up to 90% water [126]. These materials have the capability to expand by absorbing biological fluids, enabling EVs to be entrapped and providing controlled delivery [125]. Promising results have been reported using encapsulated MSC-EVs in hydrogels to promote regeneration. Mardpour et al. achieved constant EV release and resistance to degradation by using polyethylene glycol (PEG) hydrogels, in a rat hepatic regeneration model. The study showed both the feasibility of the delivery method and the pro-regenerative effect of MSC-EVs on injured liver [127]. A study by Qin et al. opted for a commercial hydrogel—HyStem-HP—to deliver EVs to promote osteogenesis in a critical size bone defect rat model, and reported that the system was responsible for bone formation enhancement [110]. This technique has also been shown efficient in cartilage regeneration, by combining EVs with a 3D device made of cartilage ECM and gelatin methacrylate hydrogel [128], and with the vesicles incorporated into a Photoinduced Imine Crosslinking (PIC) hydrogel glue [129].

Conversely, 3D polylactide (PLA) scaffolds have been investigated as potential enhancers of bone regeneration, combined with MSCs and MSC-EVs in an in vivo rat model. The study took human gingival MSCs (hGMSCs) and established several combinations to test the effects of the PLA scaffold, hGMSC-EVs and polyethyleneimine (PEI)-engineered EVs [130]. The results showed that all combinations improved bone healing, particularly the treatments with hGMSCs + 3D PLA with either hGMSC-EVs or PEI-EVs; these showed upregulation of the osteogenesis-related genes RUNX2 and Bone Morphogenic Protein (BMP) 2/4 and higher staining for Alizarin Red, a marker for calcium deposition and ECM mineralization, commonly used to evaluate osteogenesis [130]. In another study, MSC-EVs were loaded into collagen I/III sponges and employed in a rat periodontal defect model. The approach was found to promote periodontal tissue regeneration and formation of alveolar bone in vivo, with no evidence of adverse effects [131]. Further, when combined with poly (lactic-co-glycolic acid) supplemented with poly-dopamine (PLGA/pDA), adipose-derived stem cell-EVs can modulate the migration and homing of BM-MSCs to injury sites, thus increasing tissue healing [132]. A number of studies also evidence the advantages of using MSC-EVs coupled with hydrogels or scaffolds as potential therapeutic tools for skeletal regeneration [125].

**Figure 2:**
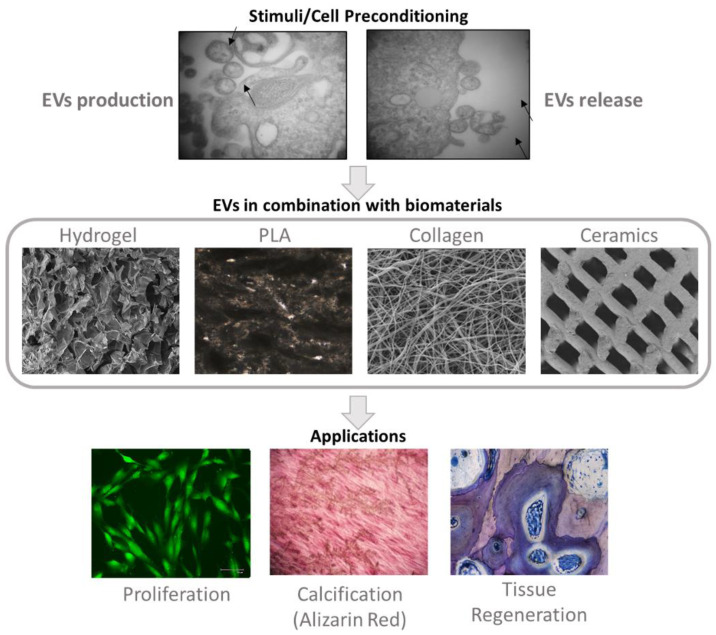
Engineering EVs and Their Applications. Cell preconditioning/engineering may increase the production and secretion of EVs, while also modulating its content in a controlled manner. These vesicles can then be delivered in combination with scaffold/biomaterials to ensure correct targeting, by conferring protection to the EVs and allowing a controlled release of their content. Thus, this strategy can lead to an improvement in tissue regeneration, through stimulation of several cell processes including proliferation, calcification and differentiation. The images depicting EVs production and release were obtained by Transmission Electron Microscopy (TEM); PLA and tissue regeneration images were obtained by the ground sections method in brightfield imaging; hydrogel, collagen and ceramics images were obtained by Scanning Electron Microscopy (SEM); the image representing proliferation was captured by confocal microscopy; the Alizarin Red staining used to detect calcification was captured in brightfield microscopy. All images belong to the authors.

Lastly, used in combination with EVs ceramics have also shown interesting results regarding bone regeneration. In general, these biomaterials possess similar compositions to the inorganic portion of bones, specifically in terms of calcium and phosphate content. Ceramics thus represent useful options for bone replacement, considering their biocompatibility, osteoconduction, and bone-cell pro-survival enhancement ability [133]. For example, β-TCP has been extensively studied in the musculoskeletal regeneration field for its elevated resorption rate and promotion of MSCs and osteoblasts’ proliferation [134]. However, the addition of EVs derived from human induced pluripotent stem cells (iPSC) differentiated into MSCs, actively improved the biomaterial’s osteoinductive activity. This combination strongly promoted MSCs’ migration, proliferation and osteogenic properties [135], revealing the important role of EVs in regenerative medicine. On that basis, EVs can also be modulated to carry specific molecules, either natural or synthetic, by cell preconditioning or engineering [136] (Figure 2).

## 6. MSC-Derived EVs Modulation through Cell Preconditioning

Preconditioning consists of exposing parent cells to specific stimuli, to enforce the expression and release of different molecules [67,125]. The focus of this review is on enhancing MSCs’ regeneration potential by modulating the EV cargo through in vitro cell culturing in a 3D environment, under hypoxia, supplemented with pharmacological agents or inflammatory cytokines (reviewed in [67]). For instance, MSCs cultured in hypoxia (1% O_2_) or anoxia (0% O_2_) have produced EVs more suited for acute myocardial infarction treatment, in in vivo rat [102] and mouse [137] models, respectively. The former study cultured BM-MSCs in hypoxia for 72 h and, after EVs injection in rats, reported increased blood flow recovery and cardiac performance, together with a reduction in infarct size, compared with controls [102]. In the latter study, MSCs were cultured overnight in glucose deprivation and then preconditioned with two cycles of anoxia and reoxygenation. Microarray analysis revealed that miR-22 was upregulated in preconditioned-MSC-EVs that, upon injection, reduced apoptosis and enhanced cardiac function after infarction [137].

Regarding the musculoskeletal system, MSCs’ preconditioning with dimethyloxaloylglycine (DMOG) was assessed in a critical-sized calvaria-defect rat model [138], a preclinical model frequently used to experimentally evaluate bone regeneration [139]. BM-MSCs were cultured with supplementation of 1000 μM DMOG for 48 h, after which the authors isolated exosomes from the culture supernatants. Then, exosomes from non-preconditioned and preconditioned MSCs were injected in the rat model, in conjugation with hydroxyapatite (HA) scaffolds. Bone regeneration in vivo was increased when rats were treated with scaffolds embedded with EVs from MSCs preconditioned with low doses of DMOG [138]. The authors demonstrated that the mechanism of action might be related to the ability of EVs released by DMOG-pretreated MSCs to enhance angiogenesis of human umbilical vein endothelial cells (HUVECs), through the downregulation of Phosphatase and tensin homolog (PTEN), which in turn activates the Akt and mammalian target of rapamycin (mTOR) (AKT/mTOR) pathway [138]. A different study used TNF-α as preconditioning—referred to as priming in the study—for AT-MSCs [140]. Lu et al. cultured the cells for 72 h with 1 ng/mL of TNF-α and evaluated the effects of the isolated EVs in human primary osteoblast-like cells (HOBs). This specific preconditioning was found to increase the pro-osteogenic and proliferative induction by EVs in HOBs, thus representing a potential alternate therapy for bone regeneration [140].

## 7. MSC-Derived EVs Modulation through Engineering

Being natural carriers, capable of avoiding immune responses, and having good stability and integrity in the blood, EVs represent an interesting approach as delivery systems. Engineered EVs can offer a combined effect between their natural cargo and externally incorporated drugs or biological material, while also directing the therapy to the intended target, thanks to the specific recognition of surface proteins by receptor cells. Current techniques focus on modifying specific portions of EVs, before and/or after their isolation: cargo editing can enhance or transform the biological function of EVs; surface and membrane editing aims to alter the expression of markers to make the vesicles traceable and/or to change their target cells, while also affecting physical and chemical properties such as solubility [141]. Loading methods with high efficiency include extrusion, sonication, and saponin-assisted loading [141]. In this case, miRNA-loaded EVs were shown to promote cartilage preservation. In OA cartilage, miR-320 expression is decreased and in concomitance an increase of MMP-13 expression was observed. Moreover, the encapsulation of miR-320 seems to regulate MMP-13, and it could be a promising strategy for cartilage protection [142]. However, some protocols can cause membrane deformation or aggregation of particles or molecules [141]. An extensive view of advantages and disadvantages is provided in the review by Man et al. [141].

Further, this semi-synthetic system is flanked by the fully synthetic approach. Nanovesicles mimicking EVs may be developed either from cultured cells (cell-derived nanovesicles—CDNs) or from individual molecules (EV-inspired liposomes—EVLs) [141,143]. The most common technique for obtaining CDNs consists of sonicating whole cells to create particles similar to vesicles, ranging in size from 50 to 200 nm; this technique affords higher quantities of particles compared to conventional EV isolation protocols [143,144]. Depending on their origin, CDNs possess the membrane composition of the parent cells, which eliminates future steps of functionalization necessary for artificial vehicles. In addition, CDNs are more stable and less toxic than synthetic, non-cell-based vehicles [143,145]. Jo et al. found that CDNs derived from embryonic stem cells can stimulate the proliferation of mouse MSCs [146]. CDNs are also reported to be effective in pathogenic situations, when injected in mice with sepsis: they inhibit common symptoms such as hypothermia and eye exudates, by upregulating IL-10. CDNs were detected throughout the whole body, namely in the lungs, kidneys, and liver [147].

EVLs are synthetic particles created to provide the minimal required element for functional delivery systems. Thus, this technology allows the controlled production of pure and specific nanoparticles, without any biological contaminants [143,148]. This “bottom-up” technology is a valid alternative to other commercial agents used for drug delivery and transfection, since it presents better storage stability, an anti-aggregation effect, and reduced toxicity [143,149]. In fact, dendritic cell (DC) derived-EV-based EVLs, containing major histocompatibility complex (MHC) Class I molecules to mimic the antigen display role to cytotoxic T cells, have been developed. These vesicles were traceable in both in vitro and in vivo settings, and contributed to adhesion and cell activation [150]. EVLs have also been used to deliver an anti-VEGF small interfering-RNA to target the A549 cell line. These EV-mimics enter A549 cells through direct fusion, and show similar uptake and efficiency compared to commercial Lipofectamine 2000 and 1,2-dioleoyl-3-trimethylammonium-propane (DOTAP) [149]. Functionalized liposomes can also be used to create scaffolds for bone regeneration, considering their features such as thermo-responsiveness, adhesiveness, bone targeting and osteoconductivity [151].

## 8. EVs Derived from MSC Differentiation

Although the multi-lineage differentiation capacity of MSCs has long been established, the biological functions of the secretome of osteoblasts, adipocytes, and chondrocytes, derived from MSC differentiation, have received little attention. However, some reported properties of the vesicles released by these cells may show potential for their establishment as therapeutic agents, particularly in the bone regeneration field.

Little is known about the direct role of adipocyte-derived EVs (Adi-EVs) in musculoskeletal processes. However, a potential role of these vesicles in bone regeneration may be inferred, considering their impact on inflammation, a key part of the healing process. Kranendonk et al. have demonstrated that Adi-EVs exert immunomodulatory effects on monocytes, aiding their differentiation into macrophages with both pro- and anti-inflammatory phenotypes [152]. Indeed, characterization of Adi-EVs reveals the presence of several cytokines, including adiponectin, TNF-α, retinol binding protein 4 (RBP4), macrophage colony-stimulating factor (M-CSF) and in particular macrophage migration inhibitory factor (MIF). This, in turn, when in contact with monocytes, induces their differentiation into adipose tissue macrophages (ATM) with their typical mixed profile, secreting both pro-inflammatory—IL-6, TNF-α and macrophage inflammatory protein-1-alpha (MIP-1α)—and anti-inflammatory proteins—IL-10 [152].

Obesity has been reported to severely impact Adi-EVs’ properties. Zhang et al. showed that Adi-EVs favor macrophage polarization into the M1 pro-inflammatory profile, using an obesity rat model. They cultured bone-marrow-derived macrophages with Adi-microvesicles from obese and standard diet mice. The results showed an increase in the M1 phenotype vs. controls, as assessed by both quantitative real-time polymerase chain reaction (qRT-PCR)—through upregulation of M1 markers TNF-α, inducible nitric oxide synthase (iNOS), and IL-12—and flow cytometry—by increased number of CD11+ cells [153]. Conversely, M2 macrophage polarization is suppressed by Adi-EVs of high-fat-diet mice, likely due to the exosomal transfer of miR-34a, which represses macrophage expression of Krüppel-like factor 4 (Klf4) [154], a transcription factor essential in the macrophage shift to the anti-inflammatory phenotype [155].

With regard to chondrocytes, there are some evidences pointing to their potential use in musculoskeletal regeneration. Chen et al. injected alginate/cartilage progenitor cell (CPC) constructs into mice, along with either chondrocyte-EVs (CD-EVs) or BM-MSC-EVs, and found that treatment with CD-EVs improved CPC migration, proliferation, and matrix formation, by upregulating SRY-Box Transcription Factor 9 (SOX-9) and collagen type II levels, while inhibiting angiogenesis. Animals treated with CD-EVs showed more favorable results than those receiving BM-MSC-EVs in terms of chondrogenesis [156]. Another study corroborated these findings by combining CD-EVs with UC-MSCs for articular cartilage repair. The study verified that CD-EVs also increased expression of the aforementioned proteins in UC-MSCs in vitro, among other proteins related to chondrocyte maturation, and enhanced knee defect healing in a rabbit model by activating autophagy [157]. However, in an OA setting, OA-derived CD-EVs may lead to IL-1β production by macrophages and negatively impact cartilage degradation and synovitis, which means that the use of autologous CD-EVs may not be suited for OA treatment [158].

Lastly, osteoblast-derived EVs (OB-EVs) have been tested with the goal of bone regeneration. Using osteogenic differentiated pre-osteoblast MC3T3-E1 cell line-derived EVs, Cui et al. induced MSC osteogenesis in vitro by modulating their microRNA profile. The authors expect these changes to impact several pathways connected with osteoblast differentiation and function, namely the Wnt, insulin, TGF-β, and calcium signaling pathways. The results also showed an increase in β-catenin, an important transcription coactivator of the Wnt pathway, through the upregulation of Ctnnb1, its encoding gene, and the inhibition of Axin1, a negative regulator [159]. The involvement of different microRNAs in osteogenesis has indeed been reported [160,161], although their delivery by OB-EVs remains largely unexplored. However, osteoblast activity can be impaired through miR-214-3p transfer from osteoclasts (bone-resorption cells) through EVs, which inhibit the bone formation process [162]. This osteoblast/osteoclast interaction by EVs also involves other players: RANKL (Receptor activator of nuclear factor kappa-Β ligand) is a TNF family member that binds to RANK receptors (Receptor Activator of Nuclear Factor kappa-Β) in osteoclast precursor cells to stimulate their differentiation into mature osteoclasts [163]. Cappariello et al. have shown that OB-EVs possess RANKL on their surface, and when in culture with osteoclasts, cell function, size, number of nuclei, and metabolic activity all increase. In RANKL^−/−^ mice, which are also commonly without expression of osteoclast marker Tartrate-resistant acid phosphatase (TRAcP), injection of OB-EVs leads to the emergence of TRAcP-positive cells, in a directly proportionate manner [164]. Osteoclast activity was also successfully inhibited in vivo, by loading OB-EVs with zolendronate and dasatinib [164]. In the light of current information, it is considered that the use of OB-EVs, either naïve or with drug incorporation, may be a promising treatment for bone related diseases—such as osteoporosis—and cancer [165].

## 9. Conclusions and Future Perspectives

MSCs have been widely studied as therapeutic tools for their ability to stimulate renewal and differentiation into specialized connective tissues (bone, cartilage, adipose tissue, muscles), as well as their capability in immunomodulation, and inducing angiogenesis, cell recruitment, differentiation, and apoptosis inhibition [18,19,20,21,22,23]. However, due to some ethical, economic, and biological disadvantages, the use of MSC-derived EVs is being increasingly studied as a replacement for the use of MSCs in regenerative medicine. These particles maintain the properties of the parent cells while avoiding some of the known drawbacks associated with cell usage. Therefore, EVs are involved in numerous physiological and pathophysiological processes: in MSDs, EVs foster tissue regeneration through the delivery of factors capable of exerting mitogenic, angiogenic and immunomodulatory effects. As explored in this review, MSCs-EVs can interact with several cell types involved in MSDs, namely osteoblasts, osteoclasts, chondrocytes, myocytes, tenocytes, immune cells, and vascular endothelial cells, being able to modulate their cellular processes towards a pro-regenerative phenotype. Despite this, in order to consider EVs as clinical options, isolation methods must be standardized, and functional knowledge regarding different cellular origin must be enhanced. EVs modulation, either by cell preconditioning or engineering, is becoming a hot topic in molecular biology that allows for more control in terms of content, mechanism of action, and yield, although standardization is lacking. The strategies to couple biomaterials and EVs can also have a big impact as a future therapy, for the two technologies can enhance each other’s properties and together represent an ideal approach for tissue healing. Lastly, using EVs derived from cell lineages that differentiate from MSCs may offer a more specific approach as targeted/personalized medicine, although focus on this field is still in its early days.

This review has explored the potential of MSC-EVs—and EVs originated from MSC-derived cell types—in several MSDs. In conclusion, the promising in vitro and in vivo studies indicate that the development of new therapies using MSC-EVs and EV modulation/engineering techniques, most notably in conjugation with biomaterials, will in the future erupt into the field of musculoskeletal regenerative medicine.
